# Enteroviruses Activate Cellular Innate Immune Responses Prior to Adaptive Immunity and Tropism Contributes to Severe Viral Pathogenesis

**DOI:** 10.3390/microorganisms13040870

**Published:** 2025-04-10

**Authors:** Jonathan A. Coffman

**Affiliations:** School of Pharmacy, American University of Health Sciences, Signal Hill, CA 90755, USA; jcoffman@auhs.edu

**Keywords:** viruses, tropism, innate immunity, adaptive immunity, antibodies, cell-mediated immunity, complement, Toll-like receptors, viral sensors, interferon, cytokines

## Abstract

Numerous innate immune mechanisms have been shown to be activated during viral infections, including pattern recognition receptors (PRRs) functioning outside and inside the cell along with other sensors promoting the production of interferon and other cytokines. Innate cells, including NK cells, NKT cells, γδ T cells, dendritic cells, macrophages, and even neutrophils, have been shown to respond to viral infections. Several innate humoral responses to viral infections have also been identified. Adaptive immunity includes common cell-mediated immunity (CMI) and humoral responses. Th1, Th2, and Tfh CD4+ T cell responses have been shown to help activate cytotoxic T lymphocytes (CTLs) and to help promote the class switching of antiviral antibodies. Enteroviruses were shown to induce innate immune responses and the tropism of the virus that was mediated through viral attachment proteins (VAPs) and cellular receptors was directly related to the risk of severe disease in a primary infection. Adaptive immune responses include cellular and humoral immunity, and its delay in primary infections underscores the importance of vaccination in ameliorating or preventing severe viral pathogenesis.

## 1. Introduction

The *Enterovirus* genus belongs to the *Picornaviridae* family [1], a large family composed of 159 species grouped into 68 genera (https://www.picornaviridae.com/ (accessed on 7 February 2025)). Enteroviruses are non-enveloped, positive-sense, single-stranded RNA viruses that replicate according to Group IV of the Baltimore designation [2] and include 15 species with enteroviruses A–D and Rhinovirus A–C associated with human disease [3,4,5]. Although the viruses belong to the same genus, the associated diseases are strikingly different. Some of the clinical diseases include polio [6]; hand, foot, and mouth disease (HFMD) [7,8]; hemorrhagic conjunctivitis [9]; gastroenteritis [10]; sudden infant death syndrome (SIDS) [11]; pneumonia [12]; and the common cold [13]. The icosahedral capsid of the non-enveloped virus has been shown to attach to distinct cellular receptors (Table 1), establishing the tropism of an enterovirus for cell types and influencing pathogenesis [14]. The purpose of this review is to evaluate five different enteroviruses and summarize how the innate immune response, the delayed adaptive immune response, and the tropism of the respective viruses contribute to viral pathogenesis, underscoring the importance of vaccination to ameliorate or prevent severe disease.

## 2. Innate Immune Responses to Viral Infections

Physical barriers are considered to be the first line of defense against pathogens and are commonly recognized as components of the innate immune response. Anatomical structures and systems also have numerous innate barriers that may be chemical or physiological in nature, and within the structures and systems, there are molecular and cellular components of innate and adaptive immunity in operation (Table 2). Where viruses enter the body—portals of entry—and the surrounding resident tissues and cells have been shown to influence the immune response, host symptoms, and disease progression. A number of reviews and studies have identified innate and adaptive immune responses in the respiratory tract [15]; gastrointestinal tract [16]; blood [17]; lymph [18,19]; nervous system [20,21]; integumentary system [22,23]; and urogenital tract [24].

## 3. General Innate Immune Responses to Viral Infections

After the first Toll-like receptor (TLR) was identified in *Drosophila* as a pattern recognition receptor (PRR) against a pathogen, researchers sought to identify the human TLRs that act as sensors for viral molecular patterns. TLR3, which recognizes viral dsRNA, was found in the endolysosomal plasma membrane and endoplasmic reticulum; TLR7, which recognizes bacterial and viral (GU-rich) ssRNA, was also found in the endolysosomal plasma membrane and endoplasmic reticulum along with the cell membrane; TLR8, which recognizes bacterial and viral (GU-rich) ssRNA, was found in the endolysosomal plasma membrane and endoplasmic reticulum; and TLR9, which recognizes viral and bacterial unmethylated cytosine phosphate guanine-dideoxy nucleotide (CpG) DNA and DNA:RNA hybrids, was again found in the endolysosomal plasma membrane and endoplasmic reticulum [25]. TLR3, 7, and 8 were known to induce the production of interferon (INF) proteins that would—in turn—bind to receptors on neighboring cells, resulting in an antiviral state that consisted of an overall decrease in protein production in addition to the activation of cells of the immune system. A total of 21 types of human IFN were identified and classified as type I (13 subtypes), II (IFN-γ), or III (antiviral defense of the mucosal epithelium) [26].

The retinoic acid-inducible gene 1 product (RIG-I) and melanoma differentiation-associated protein 5 (MDA5) were characterized as the cytoplasmic counterparts of the viral TLRs and were referred to as RIG-I-like receptors (RLRs); when activated, they were shown to increase the production of type I and type III IFNs [27]. Nod-like receptors (NLRs) and cyclic GMP-AMP synthase (cGAS) were also identified as viral sensors that monitor the cytoplasm for cell damage or pathogenic invasion, and activated cGAS resulted in the activation of the cGAS-STING pathway, leading to the production of interferons [28]. Protein kinase R (PKR) has also been shown to detect viral replication within the host cell and interfered with viral replication by phosphorylating eIF2α and halting protein synthesis [29]. The production of interferons has also been reported to induce the expression of the oligoadenylate synthetase (OAS) proteins that activate the endoribonuclease RnaseL, which degrades viral and cellular RNA to control viral infection [30]. The NALP3 inflammasome [31] and the AIM2 inflammasome [32] complexes were also shown to contribute to virally induced inflammation in response to RNA viruses and DNA viruses, respectively. Following viral detection by the sensors, inflammatory mediators attract other immune cells to the site of infection.

Historically, natural killer (NK) lymphocytes were recognized as a cellular first line of defense against virally infected cells [33]. Virally infected cells that have decreased expression of MHC1 proteins or those that secrete interferons are engaged by NK cells that degranulate and destroy the virally infected cells in an antibody-independent manner [34]. Natural killer T (NKT) cells have been found to be present in many tissues throughout the body, most abundantly in the liver, spleen, bone marrow, and thymus, and were also found in moderate numbers in the lungs, skin, and intestines [35]. NKT cells have been shown to recognize lipid antigens presented by CD1d molecules, allowing them to detect signs of infection or cellular stress, in turn producing cytokines such as IFN-γ. In contrast to NKT cells, γδ T cells are enriched in the skin, lungs, and intestines, and were found to participate in immune surveillance, with the ability to release large amounts of cytokines in addition to playing several roles in tissue physiology [36].

Phagocytic cells, which include macrophages (Mφs) and plasmacytoid dendritic cells (pDCs), are known to express TLRs differentially. Macrophages express high levels of TLR2 and TLR4, while pDCs mainly express TLR7 and TLR9 [37]. Tissue-resident dendritic cells (DCs) have been shown to be central in capturing and displaying viral antigens and also contribute to the development of immune memory [38]. Thus, DCs were shown to bridge the innate immune response with the adaptive immune response. Conventional or classical dendritic cells (cDCs) are grouped into two subsets, and the cDC1 subset has been shown to be superior in their ability to cross-present antigens to activate CD8+ T cells that play a direct role in eliminating viruses [39]. Additionally, cDC1 was found to be directly related to Th1 helper T cell responses (see the adaptive immune response below). Neutrophils—historically associated with bacterial infections—have also been shown to participate in antiviral responses through the disruption of viral envelopes, phagocytosis of antibody-coated viruses and cellular complexes, secretion of extracellular traps composed of DNA, and activation and regulation of other cells [40].

Described as part of the humoral branch of innate immunity, the complement system—a series/cascade of protein molecules—has been shown to be activated through three main pathways: the classical pathway activated by antibodies bound to viral surfaces; the alternative pathway activated directly by the viral surfaces of other factors in the absence of antibodies; and the lectin pathway activated by the binding of mannose-binding lectins (MBLs) to mannose residues on viral surfaces [41]. The antiviral effects of complement include several outcomes. Complement proteins, such as C3b, have been shown to bind to viral particles, and phagocytes have C3b receptors, resulting in enhanced phagocytosis of the C3b-bound antigen. Complement components have also been shown to directly interfere with viral attachment to host cells. Additionally, the formation of the membrane attack complex (MAC) results in cell lysis of the virally infected cell, and complement activation generates inflammatory mediators that recruit immune cells to the site of infection, promoting adaptive immune responses [42]. Other innate humoral proteins thought to be ancestral precursors of antibodies include pentraxins, collectins, and ficolins, which have been shown to activate complement and assist in the opsonization of pathogens [43].

Following the detection of viruses through pattern recognition receptors and the release of cytokines by infected tissues and immune cells, acute-phase proteins are known to be produced and released by the liver, amplifying systemic innate immunity and influencing adaptive immunity. Acute-phase proteins produced by the liver and other cells responding to viral infections have been shown to include α1-AT, SAP (serum amyloid P component), PTX3, SAA (serum amyloid A), PTX1 (C-reactive protein), MBL (mannose-binding lectin), and others out of the approximately 200 acute-phase proteins produced in the body [43].

## 4. Chemokines and the Homing of Innate Immune Cells

Chemokines are a type of cytokine that assist in the migration of immune cells—both innate and adaptive—to the site of inflammation or infection, playing important roles throughout the duration of an infection. Approximately 50 endogenous chemokine ligands and 20 G protein-coupled seven-transmembrane signaling receptors have been identified and grouped into four different sub-families: CC, CXC, CX3C, and C [44,45]. Viral infections have been shown to increase the expression of several of the chemokine ligands: CCL2, CCL3, CCL4, CCL5, CCL7, CCL8, CCL13, CXCL10, and CXCL11 [46,47]. Many of these ligands will bind to multiple chemokine receptors expressed on numerous cell types, and CX3CL1 has been found to be unique in that it bound to the CXCR3 receptor only [44]. Regionally infected cells produce chemokine gradients that affect the “stickiness” of endothelial cells in proximity to the site of a viral infection, facilitating the migration of other cells and serum proteins [47].

## 5. Adaptive Immune Responses to Viral Infections

The adaptive immune response has historically been described as the collective activities of B and T cell-mediated immunity (cellular) and humoral immunity (antibodies). In the adaptive immune response, differentiated, naïve T lymphocytes have been shown to mature and respond to a viral infection as both cytotoxic T cells (CTLs, CD8+) and helper T cells (CD4+). In a previous study, CTLs were known to be activated through a cross-priming mechanism via dendritic cells (DCs) and MHC1 presentation [48], and subsequently, activated CTLs would detect viral antigens through MHC1 presentation on the surface of infected cells, allowing the CTLs to destroy the “viral factories” (host cells). A subset of the CTLs became memory cells, providing future protection against the virus that previously caused the infection. Naïve helper T cells were shown to work in conjunction with MHC2 molecules expressed on antigen-presenting cells (DCs, macrophages, and B cells), and the subsets of activated helper T cells have been classified as Tfh (follicular helper), Th1, Th2, Th17, and Tregs [49]. Following intracellular innate immune responses, a multi-protein complex called an inflammasome was found to form and the inflammasomes not only contributed to inflammation but also helped to promote the differentiation of naïve CD4+ T cells into Th1, Th2, and Th17 subsets [50]. Th1 responses were found to be directed against intracellular infections such as viral infections, but redundant signals from many pathogens were found to also activate Tfh responses [51]. Interleukin 12 (IL-12) was found to be a common signal for the Th1 and Tfh responses, whereas interleukin 4 (IL-4) was found to direct the Th2 response against a helminth or contribute to an allergy, and interleukin 1 (IL-1) and interleukin 6 (IL-6) were found to promote the antibacterial response of the Th17 helper T cells.

Antibodies generated during viral infections have been shown to neutralize viral progeny, activate the classical complement pathway, and promote antibody-mediated opsonization. IgM—a potent activator of complement C1q—was shown to be the first made antibody in response to a viral infection [52], and cryo-atomic force microscopy confirmed the crystallographic prediction that the molecule exists as a non-planar, pentameric or hexameric, mushroom complex with its central region protruding away from antigen-binding domains [53]. The J chain, as part of the pentameric IgM, facilitates the secretion of IgM in the mucosa by interacting with the polymeric immunoglobulin receptor (pIgR) on the basolateral surface of mucoepithelial cells, allowing its transport through the cell into the lumen [54]. Polymeric IgA joined with a J chain was also shown to interact with the pIgR and transcytose epithelial cells and was identified as the predominantly secreted immunoglobulin in the airway epithelium working in conjunction with the finely tuned mucous blanket and the “beating” of the cilia [55]. The type of antibody produced was also found to be significant relative to the pathogenesis of a virus, and—in an ideal outcome—B cells could be influenced to undergo class switching to produce the appropriate antibody relative to the virus’s disease progression [56]. For example, the production of circulatory IgG in the bloodstream would help prevent the spread of a blood-borne virus to other organs, whereas the production of secretory IgA (SIgA) would be beneficial in the lumen of the gastrointestinal tract to block viral attachment to mucoepithelial cells.

Dendritic cells are constantly active in the gut sampling antigens and have been shown to be key regulators in the production of secretory IgA in the gut with their direct roles in Peyer’s patches, where they have been shown to induce Tregs to suppress immune responses against some of the antigens, but Tfh cells were also induced to promote the class switching of the IgM-producing B cells to IgA-producing B cells and their migration into the intestinal lamina propria [57]. cDC1s were shown to be central in the cross-presentation of rotavirus antigens to CD8+ T cells, and a recent report indicated that the cross-presentation of an antigen could happen independently of TLR3 in adult mice [58].

## 6. Immune Responses to Enterovirus A71 (EV-A71)

Following the binding of VP1 to the scavenger receptor class B member 2 (SCARB2) or selectin P ligand (PSGL-1) [59], human intestinal epithelial cells were shown to increase the gene expression of TLR7 and TLR8 [60], and their activation resulted in the downstream transcription of type-1 interferon genes. A recent study demonstrated that DDX6-associated viral RNA could stimulate a RIG-I-mediated type I IFN response [61], but other studies suggest that the cytoplasmic sensor MDA5 plays the principal role in sensing viral infection that ultimately results in the phosphorylation and activation of interferon regulatory factor 3 (IRF3), that can translocate to the nucleus and promotes the transcription of type-1 interferon genes [62].

Transcriptomic sequencing demonstrated increased transcription of TLR2 in EV-71 infected cells, and over-expression of TLR2 was shown to limit the replication of EV-71 [63]. Traditionally associated with the detection of bacteria, TLR2 has been shown to sense a SARS-CoV-2 envelope protein, contributing to pro-inflammatory cytokine release [64]. In encephalitis patients or I HFMD-alone patients infected with EV-71, the cytokine levels of IL-2, TNF-α, IL-4, IL-5, IL-13, IL-17A, IL-22, and IL-1β were significantly increased compared to healthy controls, and the encephalitis patients had even higher levels of IL-4, IL-5, IL-22, and IL-23 compared to the HFMD-alone patients [65]. It is interesting to note that increased expression of IL-4 and IL-5 has been shown to be characteristic of type 2 innate immune responses against parasites [66], and IL-22 and IL-23 have been linked to chronic inflammation and the activation of Th17 cells [67]. A recent study targeting the inhibition of the NLRP3 inflammasome through the VIM-ERK-NF-kb signaling pathway also indicated the importance of the innate immune response in complicated EV-A71 infections involving severe CNS disease. In glioblastoma cells, vimentin (VIM) was shown to promote ERK phosphorylation, leading to the activation of NF-κB-regulated genes and NRLP3 inflammasome activation [68]. Dendritic cells (DCs) are known to reside in the respiratory and intestinal tracts, and a recent study suggested that DCs might spread the virus to the skin and CNS in the early stage of disease through a heparin sulfate transmission mechanism that is inhibited by soluble heparin [69].

Astrocytes are the cells in the CNS that are vulnerable to infection by EV-A71, and a mouse model study recently indicated that EV-A71 infection of astrocytes activates complement and the production of C5a anaphylatoxins, which significantly contribute to the pathogenesis of EV-A71 in cases of severe CNS disease [70].

In a recent clinical study, antibody-secreting B cells in children older than 3 years of age were predominately producing IgG within the first week of EV-A71 illness but younger children were predominately producing IgM [71]. Another study found a high rate (95.6%) of serum EV-A71-IgM in patients with severe CNS infection, suggesting the ineffectiveness of IgM alone in preventing severe disease or that the progression of the disease precedes the production of IgM [72]. However, CXCR5+PD-1++ CD4+ T cells or follicular helper T cells (T_FH_) were shown to peak in the intestinal tract in infancy and were critical for the stimulation of long-lived B cells that produce high-affinity IgG, IgA, and IgE antibodies [73], suggesting that infants do have the ability to produce class-switched antibodies, but severe disease from DC spread may occur before the production of high-affinity IgG antibodies. A mouse antibody neutralization study revealed at least two mechanisms for neutralizing monoclonal antibodies: interfering with the attachment of the virus to a cell with or without capsid destabilization [74]. Plasmablast analysis of hospitalized children with HFMD revealed that both IgM and IgG monoclonal antibodies were able to neutralize EV-A71 [75], and a recent study using zymosan as an adjuvant induced mucosal immunity to the EV-A71 vaccine with the production of IgA and memory [76]. The immune responses to EV-A71 are summarized in Table 3.

## 7. Immune Responses to Coxsackievirus B3 (CVB3)

A three-stage pathogenesis that includes viral myocarditis, autoimmune myocarditis and dilated cardiomyopathy has been linked with CVB3 infection, which involves both innate and adaptive immune responses. Previous studies have shown that the pattern recognition receptors TLR3, TLR4, and TLR7 and Nod-like receptors activate type-1 interferon production and the inflammasome, contributing to viral myocarditis in CVB3-infected myocytes [77]. In the early stage of viral myocarditis, macrophages infiltrate the environment, and CVB3 capsid proteins have been shown to trigger the NLRP3 inflammasome, indicating a contributing role of macrophages to the pathogenesis of viral myocarditis [78]. In a murine model, infected neutrophils have also been implicated in this pathogenesis, with their release of myeloperoxidase and other enzymes [79] and their ability to form oxidative stress-mediated neutrophil extracellular traps (NETs) further contributing to inflammation and cellular damage [80]. Natural killer cells have also been implicated in CVB infection, clearance, and disease [81].

Recent work in Diversity Outbred (DO) mice with a live attenuated CVB3 vaccine virus demonstrated the effectiveness of IgG virus-neutralizing antibodies that could lead to a human vaccine to prevent CVB3 myocarditis [82]. Viral RNA was not detected in the heart or pancreas of CVB-challenged mice that had been vaccinated. In another CVB3 mouse model, autoreactive T cells generated during a CVB3 infection was able to cause myocarditis in naïve mice [83]. Thus, pre-formed neutralizing antibodies generated from vaccination would prevent both viral infection and subsequent autoimmune cellular responses.

## 8. Immune Responses to Echovirus 11 (E11)

E11 initially infects epithelial cells in the intestinal tract by attaching to the FcRn, triggering innate immunity in the gut, similarly to other enteroviruses, but E11 can quickly spread to other organs including the liver, pancreas, and CNS [84]. Mouse models of E11 demonstrated the importance of type I IFNs in the control of E11 replication and preventing its spread to other organs [85]. It was also found that FcRN expression was elevated in monocytes, tissue-resident macrophages, dendritic cells (DCs), and neutrophils—motile cells of innate immunity [86]. A clinical isolate of E11 from a neonate with necrotizing hepatitis revealed activation of the NLRP3 inflammasome, secretion of IL-1β, and cell death in mouse liver cells and macrophages [87].

The production and secretion of dimeric IgA in the gut mucosa have been shown to be critical in the control of enteroviruses, and patients with deficiencies in immunoglobulin production were at risk of developing neurological complications [88]. Maternal transfer of antibodies is known to protect neonates against enteroviruses through passive immunity [89], but maternal seropositivity rates for E11 were shown to be quite low in one regional study [90].

## 9. Immune Responses to Poliovirus (PV)

The polioviruses (PV-1, -2, and -3) are non-enveloped +ssRNA virus and members of Picornaviridae, a family that also includes the human rhinoviruses but whose pathogenesis is far removed from that of a respiratory virus. While a rhinovirus is acid-labile and destroyed in the gut, poliovirus infects epithelial cells of the gut and other cells expressing CD155, including neurons, leading to the paralysis associated with polio infections [91]. A recent study revealed that poliovirus predominately induces type III IFNs through TLR3/IRF1 signaling in intestinal epithelial cells, where TLR3 senses viral dsRNA and IRF1 is translocated to the nucleus to promote the expression of IFN-*γ* [92]. Intestinal villous microfold cells (M cells) of the GALT express CD155, which facilitates the endocytosis of the poliovirus into the M cells [93]. One study posited that the production of high levels of type 1 IFN prevents the replication of the virus and its spread to the blood and ultimately the central nervous system [94].

Viral antigen uptake and presentation to T cells and B cells activate adaptive immunity to the poliovirus and, in the case of mucosal immunity, induce the secretion of dimeric IgA, neutralizing the virus in the lumen of the gut [88]. Since oral polio vaccines—that do induce the production of secretory IgA—are prone to recombination with attenuated strains of polio and other enteroviruses, there has been a push to support new formulations of inactivated polio vaccines that promote the production of IgG in the bloodstream to prevent wild-type polio spreading from the gut to the blood to the central nervous system [95].

## 10. Immune Responses to Human Rhinovirus (HRV)

The respiratory tract is known to be the portal of entry for many viruses, with an incredibly large number of viruses associated with localized infection and disease [96]. Regionally divided into three areas, the upper respiratory tract (URT) was shown to be immunologically surveyed by the nasal-associated lymphoid tissue (NALT) and the cervical lymph nodes; the lower respiratory tract (LRT) with the bronchial-associated lymphoid tissue (BALT) and mediastinal lymph nodes; and the gas exchange area abundant with alveoli [97]. Each region harbors resident immune cells and is accessible to circulating immune cells during times of inflammation. Plasma cells in the lamina propria underlying the mucosa of the URT and LRT produce secretory IgA (SIgA) and IgM that bind to the pIgR, facilitating the release of polymeric immunoglobulins into the lumen, in turn contributing to the composition of the mucous blanket [98]. Early studies in mice suggested that IgG was the dominant antibody protecting the gas exchange region of the respiratory tract [99]. Other reports indicated that alveolar epithelial IgG transport most likely occurs via FcRn-mediated transcytosis and that pIgR-mediated secretion of pIgA/IgM into the alveolar lining fluid supports the function of alveolar macrophages [100].

Over 160 types of human rhinoviruses (HRVs), non-enveloped +ssRNA viruses, and viruses designated as species A, B, and C in the family Picornaviridae have been reported to cause human diseases, ranging from asymptomatic infections, common colds, lower respiratory infections in infants, asthma among children and adults, and pneumonia in immunocompromised patients [101,102]. A striking revelation was that HRV illness during infancy gave rise to childhood wheezing [103], and that wheezing illnesses in early life predicted asthmatic outcomes [104].

Asthma has been strongly associated with a Th2-dominant immune response where Th2 cells produce interleukin 4 (IL-4) that, in turn, promotes the production of IgE by B cells that have undergone class switching following antigenic challenge (sensitization); however, the IgE immunoglobulin binds to receptors on mast cells, and following secondary exposure to the antigen, the mast cell releases histamine in an anti-helminthic response rather than an antiviral response [105]. Th2 cells also produce IL-5, which promotes eosinophil recruitment and activation in the airways, contributing to damage, and Th2 cells releasing IL-13 stimulates mucus production by goblet cells, contributing to airway obstruction [106].

Rhinovirus innate immune response studies demonstrated that TLR2 was able to recognize HRV viral proteins, and that TLR7 and TLR8 recognized internal HRV ssRNA. Upon replication, dsRNA was generated and recognized by the cytosolic sensor MDA5, promoting the production of type I interferons [107]. Rhinovirus infection was also shown to result in the harmless recruitment of STING to the replication organelles (ROs)—where positive-strand RNA viruses concentrate their viral RNA-synthesizing machinery [108]—indicating that the RV interferon response was independent of the canonical cGAS-STING pathway and that RV infection might interfere with the sensing of other DNA viruses [109].

Serological analysis of human plasma samples revealed the generation of cross-neutralizing antibodies with modest cross-reactivity limited to group types [110]. Nonetheless, rhinovirus clearly induced the production of high-affinity, class-switched antibodies.

## 11. Discussion

Although viruses can be grouped into families and categories based on molecular characteristics, an understanding of the tropism of a virus for cells and tissues and its access to human anatomical systems provides better information on how the innate and adaptive immune system will respond and that understanding also provides more effective strategies for the development of a vaccine. For example, the production of secretory IgA in the mucus blanket was shown to be effective for the neutralization of viruses that can infect the respiratory or gastrointestinal tract, whereas circulatory IgG would be more beneficial in neutralizing a virus that uses the bloodstream (viremia) to spread to another organ or system (e.g., HBV and poliovirus) after bypassing innate physical barriers.

Enteroviruses replicate inside a cell in a similar fashion, but the interaction of the viral capsid proteins with specific cellular receptors establishes the tropism of the virus for cells, tissues, and organs expressing the receptor [111]. Thus, the tropism of a virus influences the pathogenesis of a virus, and the ability of the viruses to interfere with innate immune mechanisms in cells at the portal of entry [112] allows the virus to spread to other tissues in a primary infection since neutralizing antibodies have yet to be formed. Relative to the pathogenesis of each enterovirus, the obvious strategy is to generate high-affinity neutralizing antibodies that have undergone class switching to secretory IgA or circulatory IgG.

Since rhinoviruses replicate in the respiratory tract and spread locally, an effective vaccine—such as recombinant polio–rhinovirus chimera—would engage dendritic cells in the stimulation of CD4+ T cells, resulting in sIgA producing plasma cells and the establishment of tissue-resident memory cells throughout the respiratory tract [113]. Vaccination would help prevent severe disease but also steer the immune response to a Th1 response away from the asthma-related Th2 responses.

Poliovirus vaccines—the oral polio vaccine (OPV) and the inactivated polio vaccine (IPV)—were developed to induce the production of sIgA in the gut and circulatory IgG in the blood and lymph, respectively, and in 2016, the World Health Organization (WHO) recommended the administration of one IPV preceding the trivalent OPV in resource-poor countries to prevent genetic reversions that could lead to vaccine-associated paralytic polio when administrating the OPV alone [114]. The OPV was later changed to a bivalent formulation reflecting the two types in circulation, and a new OPV was also generated with genome-wide modifications [115]. A dual approach of using an attenuated virus vaccine along with either inactivated viruses, viral-like particles, viral subunits, vectors, or DNA/RNA vaccines might also be effective in preventing myocarditis and complications due to Coxsackievirus B3 infection and CNS disease due to enterovirus A71 infection [116]. Inactivated enterovirus A71 vaccines alone have already demonstrated their effectiveness in reducing the incidence of HFMD in Kunming, China [117].

Echovirus 11 is problematic among neonates, and the importance of maternal antibodies providing passive immunity was referenced above. Accordingly, an inactivated vaccine administered to pregnant women in serial doses—if necessary—would generate IgG, which would provide passive immunity to the newborn [118,119,120]. A recent outbreak of a new E11 variant in France resulted in the deaths of seven out of nine children due to liver failure, where the neonates developed symptoms between the 3rd and 5th day of life; all tested mothers were E11-positive [121].

Enteroviruses are ubiquitous and diverse and cause a wide range of diseases. Effective vaccine development will take into consideration viral transmission, portals of entry, and the spread of the virus to permissive tissues and organs along with the antigenic determinants of the viral capsid that generate neutralizing antibodies.

## Figures and Tables

**Table 1:** Enteroviruses and their respective viral attachment proteins and receptors and pathogenesis. VP (viral protein), SCARB2 (Scavenger Receptor Class B Member 2), PSGL-1 (P-Selectin Glycoprotein Ligand-1), KREMEN1 (Kringle-Containing Transmembrane Protein 1), CAR (Coxsackievirus and Adenovirus Receptor), DAF (Decay-Accelerating Factor), FcRn (neonatal Fc receptor), CD155 (Cluster of Differentiation 155), ICAM-5 (Intracellular Adhesion Molecule 5), ICAM-1 (Intracellular Adhesion Molecule 1), LDLR (Low-Density Lipoprotein Receptor), CDHR3 (Cadherin Related Family Member 3).

Enterovirus (EV)	Viral Attachment Protein/Receptor	Pathogenesis
Enterovirus A71 (EV–A71)	VP1 and VP2 capsid proteins/SCARB2, PSGL-1	Hand, foot, and mouth disease (HFMD), brainstem encephalitis, aseptic meningitis, acute flaccid paralysis, and pulmonary complications
Coxsackievirus A6 (CV–A6)	VP2/KREMEN1 (KRM1)	HFMD, herpangina, conjunctivitis, and pneumonia
Coxsackievirus B1 (CVB1)	VP1/CAR, DAF	Type 1 diabetes (T1D), pleurodynia, aseptic meningitis, and neonatal sepsis
Coxsackievirus B2 (CVB2)	VP1/CAR, DAF	Acute myocarditis, aseptic meningitis, and acute meningoencephalitis
Coxsackievirus B3 (CVB3)	VP1/CAR, DAF	Myocarditis and heart failure
Coxsackievirus B4 (CVB4)	VP1/CAR, DAF	T1D
Coxsackievirus B5 (CBV6)	VP1/CAR, DAF	HFMD, aseptic meningitis, viral encephalitis, acute flaccid paralysis (AFP), myocarditis, and T1D
Coxsackievirus B6 (CBV6)	VP1/CAR, DAF	Myocarditis, pericarditis, meningitis, and pancreatitis
Echoviruses (E6, E9, E11, EV30)	VP1/FcRn	Aseptic meningitis, encephalitis, fever, respiratory illness, and gastrointestinal
Enterovirus C (Poliovirus 1,23)	VP1 Canyon/CD155	Poliomyelitis
Enterovirus C95 (EV–C95)	VP1	AFP
Enterovirus D68	/Sialic acid, ICAM-5, Sulfated glycosaminoglycans	Pneumonia and acute flaccid myelitis (AFM)
Rhinovirus A (80 serotypes)	VP1 Canyon/ICAM-1, LDLR, CDHR3	Rhinitis, otitis media, sinusitis, mainly upper respiratory tract infections (URTIs) with lower respiratory tract infections (LRTIs) in vulnerable populations
Rhinovirus B (32 types)	VP1 Canyon/ICAM-1, LDLR, CDHR3	Rhinitis, pharyngitis, bronchiolitis, bronchopneumonia, and asthma
Rhinovirus C (C1–C57)	VP1 Canyon/ICAM-1, LDLR, CDHR3	URTIs to serious LRTIs, bronchiolitis, pneumonia, wheezing, and asthma

**Table 2:** Select anatomical regions with their physiological and specialized structures along with known innate and adaptive immune responses.

Anatomical System/Region	Physiological Structures and Processes	Innate Immunity	Adaptive Immunity
Upper respiratory tract	Mucous blanket, cilia, turbinates, rhinorrhea, microbiota, nasopharynx-associated lymphoid tissue (NALT)	α, β-defensins, cathelicidin LL-37, mucins, complement, surfactant-associated proteins (SPs), SP-A and SP-D, PRRs, cytokines, NK cells, neutrophils, interstitial macrophages, dendritic cells, innate lymphoid cells (ILCs)	Predominantly secretory IgA and IgM, CD8+ T cells, CD4+ T cells
Lower respiratory tract	Mucous blanket, cilia, branched bronchi and bronchioles, mucociliary clearance, coughing, lung microbiota, bronchus-associated lymphoid tissue (BALT) in the LRT	α, β-defensins, cathelicidin LL-37, mucins, complement, SP-A and SP-D, PRRs, cytokines, NK cells, neutrophils, interstitial macrophages, dendritic cells, ILCs	Predominantly secretory IgA and IgM, CD8+ T cells, CD4+ T cells
Gas exchange	Distal location to upper respiratory tract, surfactant	α, β-defensins, cathelicidin LL-37, SP-A and SP-D, PRRs, cytokines, NK cells, neutrophils, mast cells, alveolar macrophages, dendritic cells	Predominately IgG, CD8+ T cells, CD4+ T cells
Oral cavity	Saliva, mastication, oral microbiota, deglutition	Lysozyme, α, β-defensins, lactoferrin, mucins, salivary agglutinin	Predominantly secretory IgA, CD8+ T cells, CD4+ T cells
Esophagus	Mucous blanket, saliva, peristalsis, microbiota	α, β-defensins, cathelicidin LL-37, PRRs, Cytokines, NK cells, neutrophils, interstitial macrophages, dendritic cells, Lysozyme, lactoferrin, mucins, salivary agglutinin (SAG), NK cells, NK T cells and IFN-γ,	Secretory IgA and IgM, IgG, CD8+ T cells, CD4+ T cells
Stomach	Mucous, low pH, digestion, gastric epithelial cells	Gastric acid, pepsin, ribonucleases, interferons, mucins, α, β-defensins, cathelicidin LL-37	Secretory IgA, CD8+ T cells, CD4+ T cells
Small intestine	Mucous blanket, peristalsis, Peyer’s patches, isolated lymphoid follicles, mucosal-associated lymphoid tissue (MALT), intestinal intraepithelial cells, differentiated enterocytes and Paneth cells, pancreatic secretions, gall bladder secretions, mesenteric lymph nodes, small intestinal microbiota	α, β-defensins, cathelicidin LL-37, PRRs, cytokines, ribonucleases and proteases, SAG, bile acids, NK cells, neutrophils, interstitial macrophages, dendritic cells, lysozyme, lactoferrin, mucins, Bacteriocins, Short-Chain Fatty Acids (SCFAs), exopolysaccharides, ribonucleases, bacterial-produced antiviral metabolites, γδ T cells, NK cells, NK T cells, IFN-γ	Secretory IgA and IgM, IgG, CD8+ T cells, CD4+ T cells
Large intestine	Mucous blanket, peristalsis, defecation, Peyer’s patches, Isolated lymphoid follicles, Intestinal intraepithelial cells, differentiated enterocytes and Paneth cells, pancreatic secretions, gall bladder secretions, mesenteric lymph nodes, large intestinal microbiota	α, β-defensins, cathelicidin LL-37, PRRs, cytokines, ribonucleases and proteases, SAG, bile acids, NK cells, neutrophils, interstitial macrophages, dendritic cells, lysozyme, lactoferrin, mucins, Bacteriocins, Short-Chain Fatty Acids (SCFAs), exopolysaccharides, ribonucleases, bacterial-produced antiviral metabolites, γδ T cells, NK cells, NK T cells, IFN-γ	Secretory IgA and IgM, IgG, CD8+ T cells, CD4+ T cells
Blood circulation	Smooth endothelial lining, blood pressure, bone marrow, thymus, spleen	Complement, PRRs, cytokines, macrophages, dendritic cells, γδ T cells, NK cells, NK T cells, IFN-γ	IgM, IgG, serum IgA, CD8+ T cells, CD4+ T cells
Lymphatic system	Internal, regional lymph nodes, meningeal lymphatic vessels and the glymphatic system, one-way valves	Complement, PRRs, cytokines, chemokines, lymphatic endothelial cells (LECs), macrophages, dendritic cells, γδ T cells, NK cells, NK T cells, IFN-γ	IgM, IgG, serum IgA, CD8+ T cells, CD4+ T cells, B cells
Nervous system	CSF, enosseous sequestration, meningeal layers, choroid plexus, connective tissue barriers	Complement, chemokines, astrocytes and IL-15, parenchymal microglia and non-parenchymal border-associated macrophages, dendritic cells, γδ T cells, NK cells, NK T cells, IFN-γ	IgM, IgG, serum IgA, CD8+ cells, CD4+ T cells
Epidermis/dermis	Stratum corneum, desquamation, skin microbiota, sebaceous glands, eccrine and apocrine sweat glands	Sebum, sweat, bacterial antivirals, defensins, SCFAs, Langerhans cells, keratinocytes, fibroblasts, melanocytes, eosinophils, basophils, mast cells, NK cells, γδ T cells, dermal dendritic cells	IgM, secretory IgA, IgG, IgE, CD8+ T cells; Th1, Th2 and Th17 CD4+ T cells
Mucoepithelium in the urogenital system	Mucous, desquamation into lumen, urine flow, low pH, MALT	Mucin, defensins and cathelicidins, cytokines, chemokines, complement, TLRs, macrophages, neutrophils, mast cells, NK cells, γδ T cells, dendritic cells	Secretory IgA, IgM and IgG, CD8+ T cells

**Table 3:** Comparative analysis of select enteroviruses: portals of entry, tropism, pathogenesis, innate immunity, adaptive immunity, and tissue-resident memory.

Virus	Portal of Entry	Tropism	Pathogenesis	Innate Immunity	Adaptive Immunity	TRM
EV-A71	Oral, Resp, Gastro	SCARB2, PSGL-1, HS	HFMD, CNS disease	TLR2, 7, 8, RIG-1, MDA5, DCs, complement, P-cytos	IgM, IgG, IgA, CD8+ and Th1 CD4+ T cells	MALT, GALT, spleen, circulation
CVB3	Resp, mainly Gastro	CAR, DAF	Myocarditis	TLR3, 7, 8, IFN-α and IFN-β, Mφs, DCs, complement, P-cytos	IgM, IgG, IgA, CD8+ and Th1 CD4+ T cells	MALT, GALT, spleen, heart tissue, circulation
E11	Gastro	FcRn	CNS disease	TLR3, 7, RIG-1, MDA5, IFN-α and IFN-β, NK cells, P-cytos	IgM, IgG, IgA, CD8+ and Th1 CD4+ T cells	MALT, GALT, spleen, circulation
PV	Intestinal epithelium, viremia to CNS	CD-155	Poliomyelitis, meningitis	TLR7, TLR8, RIG-1, IFN-α and IFN-β, IL-1α, IL-1b, NK cells, Mφs, complement, γδ T cells, IFN-γ, DCs, P-cytos	Th1 (Dominant) and Th2 CD4+ T Cells, CD8+ T cells, secretory IgA and IgM, circulatory IgG	MALT, GALT, bone marrow, lymph nodes, spleen, circulation
Rhinovirus	Respiratory tract, mucoepithelium	ICAM-1, LDLR, CDHR3	Rhinitis, pharyngitis, bronchiolitis, bronchopneumonia, asthma	TLR3 and TLR7/8, IFN-α and IFN-β, NK cells, Mφs, DCs, complement, P-cytos	IgM, IgG, IgA, Th1 and Th2 CD4 T cells, CD8+ T cells	Deep cervical lymph nodes, tonsils and adenoids, mucosal lamina propria, BALT

TRM (tissue-resident memory), EV-A71 (enterovirus A71), Resp (respiratory tract), Gastro (gastrointestinal tract), SCARB2 (Scavenger Receptor Class B Member 2), PSGL-1 (P-Selectin Glycoprotein Ligand-1), HS (Heparan Sulfate), HFMD (hand, foot, and mouth disease), CNS (central nervous system), TLR (Toll-like receptor), RIG-1 (Retinoic acid-Inducible Gene 1 product), MDA-5 (Melanoma Differentiation-Associated protein 5), DCs (dendritic cells), P-cytos (pro-inflammatory cytokines), Ig (immunoglobulin), MALT (mucosal-associated lymphoid tissue), GALT (gut-associated lymphoid tissue), CVB3 (Coxsackievirus B3), CAR (Coxsackievirus and Adenovirus Receptor), DAF (Decay-Accelerating Factor), INF (Interferon), Mφs (monocyte-derived macrophages), E11 (echovirus 11), FcRn (Neonatal Fc Receptor), PV (poliovirus), CD-155 (Cluster of Differentiation 155), γδ (gamma delta T cells), ICAM-1 (Intracellular Adhesion Molecule 1), LDLR (low-density lipoprotein receptor), CDHR3 (Cadherin-related Family Member 3), BALT (bronchus-associated lymphoid tissue).

## Data Availability

The published data are available from the articles referenced.
